# Localization and Ordering of Lipids Around Aquaporin-0: Protein and Lipid Mobility Effects

**DOI:** 10.3389/fphys.2017.00124

**Published:** 2017-03-02

**Authors:** Rodolfo Briones, Camilo Aponte-Santamaría, Bert L. de Groot

**Affiliations:** ^1^Computational Biomolecular Dynamics Group, Max Planck Institute for Biophysical ChemistryGöttingen, Germany; ^2^Molecular Biomechanics Group, Heidelberg Institute for Theoretical Studies and Interdisciplinary Center for Scientific ComputingHeidelberg, Germany

**Keywords:** lipid-protein interactions, annular lipids, lens aquaporin-0, molecular dynamics simulations, hydrophobic matching, lipid thinning, membrane protein lipid sorting

## Abstract

Hydrophobic matching, lipid sorting, and protein oligomerization are key principles by which lipids and proteins organize in biological membranes. The Aquaporin-0 channel (AQP0), solved by electron crystallography (EC) at cryogenic temperatures, is one of the few protein-lipid complexes of which the structure is available in atomic detail. EC and room-temperature molecular dynamics (MD) of dimyristoylglycerophosphocholine (DMPC) annular lipids around AQP0 show similarities, however, crystal-packing and temperature might affect the protein surface or the lipids distribution. To understand the role of temperature, lipid phase, and protein mobility in the localization and ordering of AQP0-lipids, we used MD simulations of an AQP0-DMPC bilayer system. Simulations were performed at physiological and at DMPC gel-phase temperatures. To decouple the protein and lipid mobility effects, we induced gel-phase in the lipids or restrained the protein. We monitored the lipid ordering effects around the protein. Reducing the system temperature or inducing lipid gel-phase had a marginal effect on the annular lipid localization. However, restraining the protein mobility increased the annular lipid localization around the whole AQP0 surface, resembling EC. The distribution of the inter-phosphate and hydrophobic thicknesses showed that stretching of the DMPC annular layer around AQP0 surface is the mechanism that compensates the hydrophobic mismatch in this system. The distribution of the local area-per-lipid and the acyl-chain order parameters showed particular fluid- and gel-like areas that involved several lipid layers. These areas were in contact with the surfaces of higher and lower protein mobility, respectively. We conclude that the AQP0 surfaces induce specific fluid- and gel-phase prone areas. The presence of these areas might guide the AQP0 lipid sorting interactions with other membrane components, and is compatible with the squared array oligomerization of AQP0 tetramers separated by a layer of annular lipids.

## 1. Introduction

The interplay between lipids and membrane proteins plays a fundamental role in biological membranes (Marsh, 2008; Lee, 2011). Our current understanding of the lipid-protein dynamics indicates that both, proteins, and lipids might undergo correlated structural changes that influence the assembly, organization, and ultimately the function of biological membranes (Killian, 1998; de Planque and Killian, 2003; Lee, 2003). These changes appear to be largely triggered by the minimization of the hydrophobic areas exposed to the aqueous solutions of the membrane proteins—or peptides—and the lipids around them, concept known as hydrophobic matching (Killian, 1998). Experimental and computational studies have shown in detail how peptides in membranes locally affect each other due to hydrophobic mismatch. A peptide might change its conformation, orientation, or oligomerization state in the membrane. In addition, the lipids might locally adapt by thickening or thinning around an embedded peptide. Recruiting of specific lipids around proteins (lipid sorting), or selective partitioning of proteins in certain lipids (rafts) are other possible mechanisms in real, complex, and multicomponent biological membranes (Piknová et al., 1993; Dumas et al., 1997; Killian, 1998; de Planque and Killian, 2003). However, the molecular determinants between integral membrane proteins and their surrounding lipids remain partially unresolved. In this work, by using a single component model membrane, we studied the effect of temperature, lipid gel-phase, and reduced protein mobility on the lipid distribution around the integral membrane protein aquaporin-0 (AQP0).

Several studies have shown how membranes are affected by the presence of peptides or proteins under different hydrophobic matching conditions. Simulations of the gramicidin A peptide in different membranes showed that its presence introduces structural and dynamic changes in and beyond the annular lipid layer (Kim et al., 2012). The KALP, WALP, and related α-helical peptides have illustrated that peptide tilting, association, lipid thinning or thickening, and specific lipid–amino acid interactions are involved in the peptide-lipid response under different hydrophobic mismatch conditions (de Planque and Killian, 2003; Kandasamy and Larson, 2006; Kim and Im, 2010; Monticelli et al., 2010). Bigger integral membrane proteins like the potassium channel KcsA were found to locally reduce the diffusion of its annular lipids (Niemelä et al., 2010). Experimental studies on bacteriorhodophsin (bR) have shown that its presence is capable of recruiting lipids (Dumas et al., 1997), and inducing temperature shifts in the phase transition of glycerophospholipids (Piknová et al., 1993). Thinning of membranes around an aquaporin-0 (APQ0) tetramer have also been reported (Stansfeld et al., 2013). In addition, several copies of membrane proteins (including AQP0) inserted in model membranes at physiological concentrations, are able to modify the membrane stiffness (Fowler et al., 2016). Mass spectrometry experiments in combination with MD simulations revealed a broad range of lipid binding affinities for membrane proteins, which critically influence their structural stability, oligomerization state, and function (Laganowsky et al., 2014; Landreh et al., 2016; Gupta et al., 2017). State of the art simulations have shown that is possible to study lipid sorting of small proteins in binary lipid mixtures (Yin and Kindt, 2012). Nevertheless, protein simulations in the presence of cholesterol considerably reduce the diffusion of the lipid components (O'Connor and Klauda, 2011), which in turn increases the simulation times necessary for equilibration and sampling, beyond the multi-microsecond scales (Baker and Abrams, 2014).

**Figure 1:**
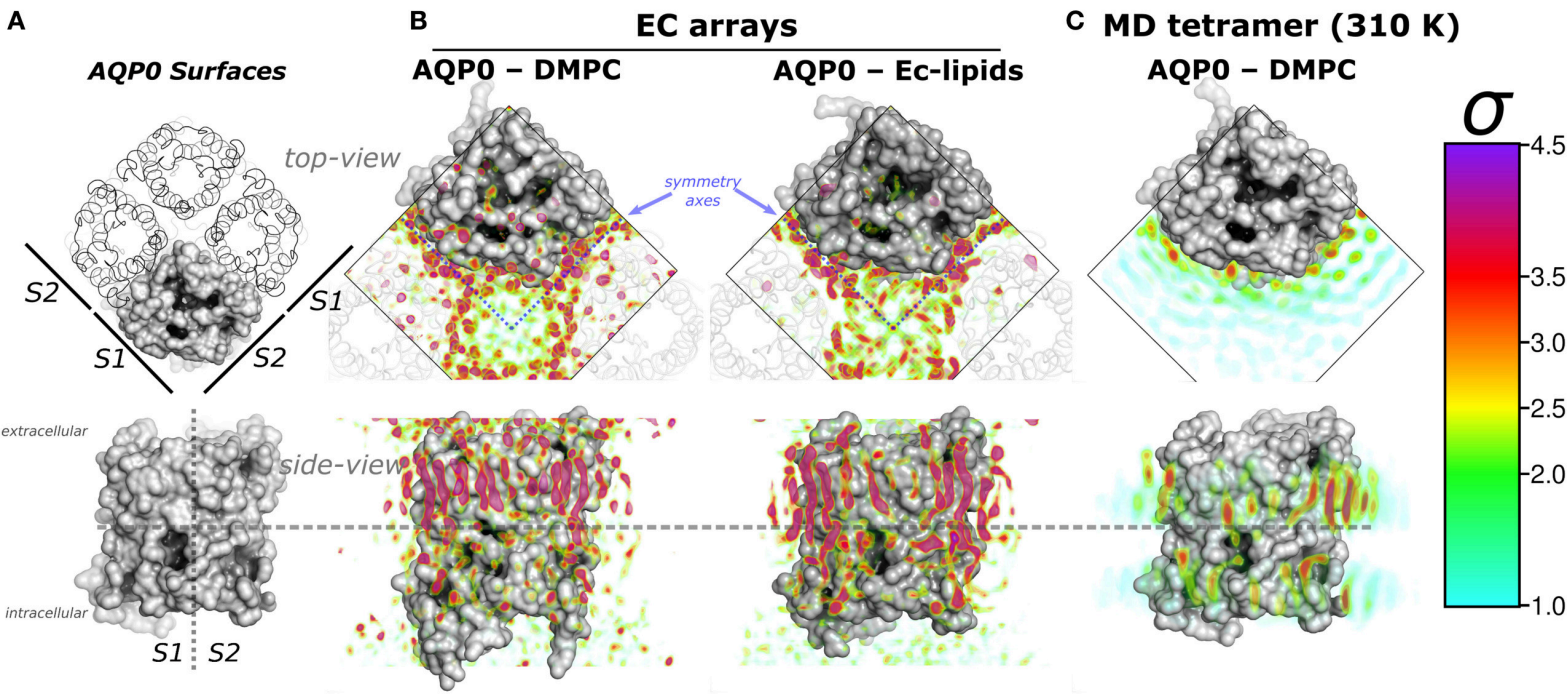
**Aquaporin-0 (AQP0) surfaces, electron crystallography (EC), and time-averaged molecular dynamics (MD) lipid density (ρ_*L*_) comparison**. Systems are shown from the extracellular side (*top-view*) and below, parallel to the membrane normal (*side-view*). **(A)** Shows an AQP0 tetramer with one of the monomers in a gray surface representation. The lipid exposed surfaces were labeled *S*1 and *S*2 (as in Aponte-Santamaría et al., 2012). The segmented lines in the *side-view* representation separate both surfaces, and the extra- and intracellular sides of an AQP0 monomer. **(B,C)** show identical volumes of normalized ρ_*L*_, corresponding to EC and MD, respectively. The ρ_*L*_ were contoured in the color-coded standard deviation (σ) scale on the right. **(B)** Shows the masked EC-AQP0 densities (excluding the protein) solved in DMPC (Gonen et al., 2004), and *E. coli* polar lipids (Hite et al., 2010). In the *top-view* perspectives, the ρ_*L*_ along the symmetry axes (blue segmented lines) contact *S*1 and *S*2 surfaces of adjacent tetramers shown as transparent ribbons. Similarly stretched acyl-chain-like ρ_*L*_ shapes are observed in the *side-view* perspective. **(C)** Shows the time- and monomer-averaged MD-ρ_*L*_ of an AQP0 tetramer inserted in a DMPC bilayer, simulated at 310 K.

AQP0 is one of the few membrane protein that provides atomistic details of protein-lipid complexes. AQP0 monomers arrange functionally as homotetramers, and are exclusively expressed in fiber lens cell membranes, where they allow water permeation and mediate intermembrane junctions (Chepelinsky, 2009). As shown by electron- (Costello et al., 1989), atomic force- (Colom et al., 2012) (AFM), and hybrid high-speed atomic force–optical microscopy (Colom et al., 2013), AQP0 is able to natively form self assembled 2D tetramer array domains. Electron crystallography (EC) (Gonen et al., 2004, 2005; Hite et al., 2010) of 2D crystal arrays of reconstituted AQP0-phospholipids have shown the molecular details of the AQP0 structure surrounded by a lipid shell (also called annular lipids) to a resolution below 2 Å. A similar single layer of the electron densities, refined as lipid molecules (ρ_*L*_), formed a compact layer of annular lipids, sandwiched between AQP0 tetramers, was observed either with the single component membrane dimyristoylglycerophosphocholine (DMPC) or with the non native *E. coli* polar lipids (Figures 1A,B). These lipids are different to the native lipid composition of the ocular lens membranes that consist mainly of sphingomylein, and a highly variable cholesterol content (Borchman and Yappert, 2010). Nevertheless, the lattice parameters of the *in vivo* AQP0 arrays (Colom et al., 2012, 2013) are identical to those of EC structures. Thus, AQP0 (in particular surrounded by DMPC lipids) constitutes an ideal system to study lipid-protein interactions. This information also indicates that AQP0 tetramer array formation is possible under different hydrophobic matching circumstances. Furthermore, it suggests that the lipid accommodation observed here for DMPC may also occur for other more physiologically relevant lipid species beyond *E. coli* polar lipids.

In the eye lens membranes most of the lipids are associated with proteins and the lipid composition varies considerably at different parts of the lens (Borchman and Yappert, 2010). These proteins include around 60% of AQP0 and 15% of connexins (Colom et al., 2012). Other important proteins are the plasma membrane Ca^+2^–ATPase, and the Na,K–ATPase, which are necessary to keep the ion homeostasis and lens clarity (Borchman and Yappert, 2010). From atomic force microscopy on lens fiber cells (Colom et al., 2012), an interaction energy of −2.7 *k*_*B*_*T* between AQP0 tetramers was estimated. Using this energy value in 2D Monte Carlo simulations gave an estimated value of 1% of free AQP0 tetramers. The native membrane composition of human fiber lens includes variable cholesterol content and longer dihidrosphingomylein lipids, which contain 60% of palmitoyl (C16:0) and 25% nervonoyl (C24:1 ω9) acyl-chains (Borchman and Yappert, 2010). Although, the *in vivo* molecular details of the AQP0–lipids interfaces is not known, it most likely to vary at different parts of the eye lens membranes, therefore the 2D-arrays found in electron crystallography (Gonen et al., 2004; Hite et al., 2010) do represent physiollogically relevant states captured by AFM (Colom et al., 2012, 2013).

Remarkably, EC- and MD-ρ_*L*_ of DMPC around AQP0 show similar localization (Aponte-Santamaría et al., 2012). These ρ_*L*_s depict 7–9 annular lipids per monomer, that show higher intensity and more straightened densities at the extracellular leaflet (Figures 1A,B). ρ_*L*_ shapes and intensities are especially similar at the *S*2 surface, where a higher ρ_*L*_ is observed by MD at room-temperature (Aponte-Santamaría et al., 2012), and also at physiological temperature (Figure 1C). The identified driving principles of the annular lipid localization around AQP0 were of protein origin, these principles were found to consist of a combination of favorable van der Waals interactions, protein–lipid surface complementarity, and protein mobility (Aponte-Santamaría et al., 2012). This evidence indicate that the protein guides the lipid distribution, and little influence on the protein is exerted by the lipids. Although, we cannot rule out the possibility that other lipids might exert structural protein changes, the two AQP0 structures, solved in different lipid environments (DMPC and *E. coli* lipids) (Gonen et al., 2004; Hite et al., 2010), were found to be highly similar (with a backbone RMSD below 1 Å). This suggests different lipid components to promote protein structural effects only to a minor extent in AQP0.

However, cryogenic temperatures and crystallographic packing might alter the protein–lipids mobility. Considering the fact that AQP0 forms similar 2D arrays in model as well as in native membranes, it is reasonable to think that the molecular determinants of the protein–lipids interactions might be also similar. In this work we want to understand how do (a) lipids shape the protein surface, and (b) protein (mobility) shape the lipid localization and ordering. For that purpose, we simulated a single AQP0 tetramer inserted in a DMPC bilayer at physiological (310 K), and at DMPC gel-phase temperature (280 K). To decouple the protein and lipid mobility effects, we induced gel-phase in the lipids, or restrained the protein.

## 2. Methods

Molecular dynamic simulations were performed with GROMACS 5.0 (Abraham et al., 2015) software package on an AQP0-DMPC system, which consisted of 4 AQP0 monomers (PDB id: 3M9I; Hite et al., 2010), 392 DMPC (C14:0) lipid molecules, water, and 150 [mM] NaCl. Using the constant particle (~ 140,000 atoms), isotermic, isobaric (1 bar) ensemble (NPT), Systems in different “mobility” conditions were pre-equilibrated. Namely: 1. *T* = 310 K (37 °C, physiological); unrestrained system, equilibration time: 100 ns. 2. *T* = 280 K (7 °C); DMPC at gel-phase (*L*_β_) and not ice-water, equilibration time: 400 ns. 3. *T* = 310 K; induced DMPC-gel by changing the water model, equilibration time: 280 ns. The modified CHARMM water model (MacKerell et al., 1998) keeps the DMPC system in the fluid phase at 310 K (*L*_α_). However, the original TIP3P water (Jorgensen et al., 1983), induces gel-phase, as it is know for C36 lipids (Pastor and MacKerell, 2011; Boonstra et al., 2016). 4. *T* = 310 K; a protein restrained system, with a reduced protein mobility was obtained by applying a force constant of *k* = 1000 *kJ*/(*nm*^2^ *mol*) on the non-hydrogen atoms, equilibration time: 170 ns. Protein (Bjelkmar et al., 2010) and lipids used CHARMM forcefields (Klauda et al., 2010). An integration time step of 2 fs was used with Verlet algorithm (Swope, 1982). The PME algorithm was used for electrostatic interactions (Essmann et al., 1995). Potential-shift with the Verlet cutoff-scheme from 0.8 to 1.0 nm was used for short range electrostatic and van der Waals interactions. The V-rescale (Bussi et al., 2007) and the Parrinello-Rahman (Nosé and Klein, 1983) algorithms were used for temperature and pressure coupling.

The lipid densities (MD-ρ_*L*_) were estimated from 280 ns MD trajectories using a modified software tool, which allows to calculate the time-averaged density of molecule(s) around e.g., a protein (Aponte-Santamaría et al., 2012). The implemented grid based approach, considered the atomic scattering factors taken from Hirai et al. (2007). The trajectories were pre-processed to account for the four-fold symmetry of AQP0 structures. The local lipid ordering was estimated with g_lomepro (Gapsys et al., 2013) in a 100 × 100 grid. The calculated lipid properties were: inter-phosphorous (P–P), and hydrophobic thicknesses, the later using the carbon-2 average position from both acyl-chains (Kim et al., 2012); the area-per-lipid (APL), and the carbon-deuterium order parameters (SCD). The interaction energies and protein fluctuations (RMSF) were estimated with Gromacs tools. The rendering of molecular images was done with the software Pymol (Schrödinger, 2015).

## 3. Results and discussion

In the EC structures, the electron densities refined as lipid molecules (ρ_*L*_) contact the two AQP0 symmetry related surfaces (termed *S*1 and *S*2) of adjacent tetramers. Representations of AQP0 surfaces and the EC ρ_*L*_ are shown in Figures 1A,B. At physiological temperature, the average MD-ρ_*L*_ of an AQP0 tetramer inserted in DMPC is shown in Figure 1C. By changing the AQP0-DMPC system mobility we want to understand how the temperature, induced lipid gel-phase, and restraining the protein contribute to the localization and ordering of unconstrained lipids around AQP0.

### 3.1. Lipids localization

**Figure 2:**
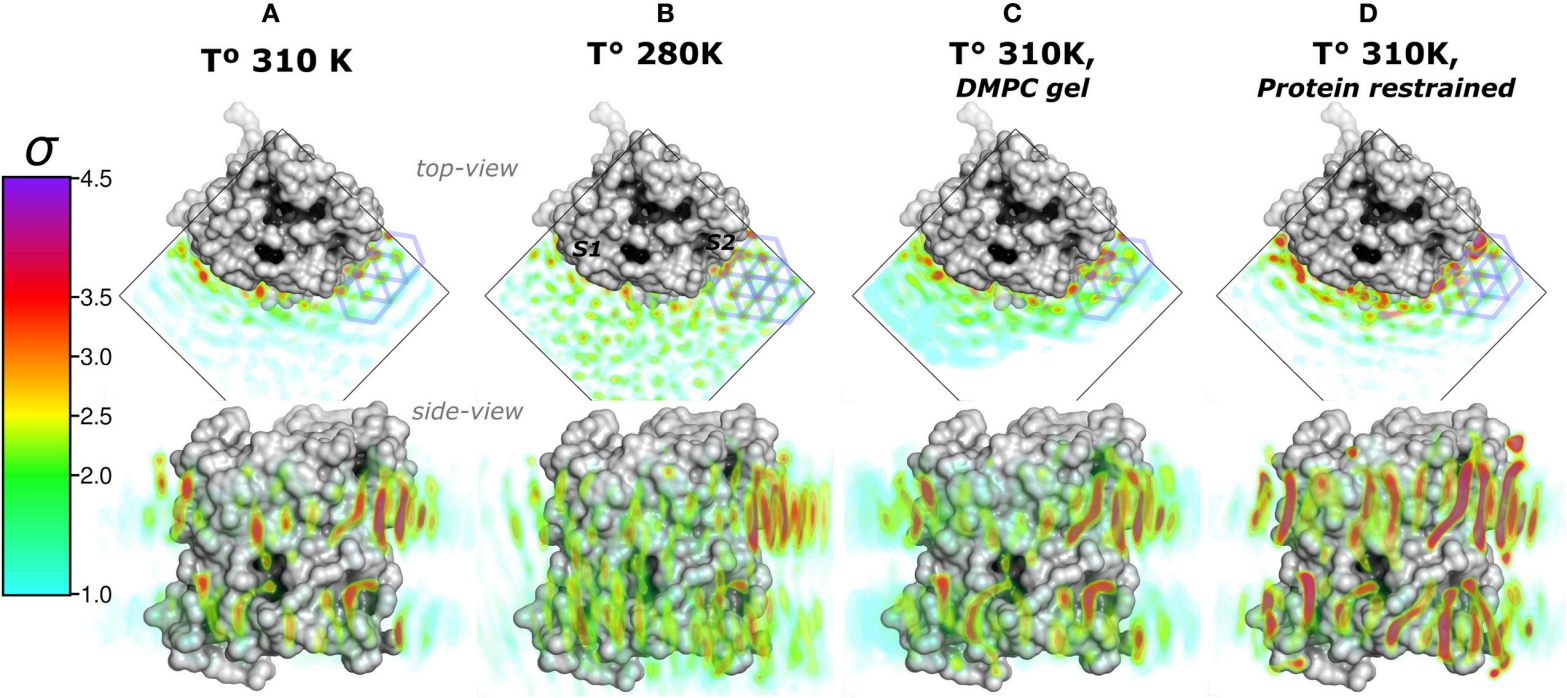
**Total lipid densities (ρ_*L*_) of AQP0-DMPC molecular dynamics simulations in different conditions: At 310, 280 K, under induced DMPC gel-phase (310 K), and restraining the protein (310 K)**. The figures show the time- and monomer-averaged densities of DMPC lipids around an AQP0 monomer in a surface representation. The densities were normalized, and shown in the σ color-scale depicted on the left. *Top-* and *side-view* perspectives of the systems are shown. The *top-view* perspective shows with blue lined hexagons the ρ_*L*_ gel-like packing observed near the *S*2 surface. **(A)** Shows the ρ_*L*_ of a AQP0-DMPC system simulated at 310 K as in Figure 1C. **(B)** Shows the ρ_*L*_ of a AQP0-DMPC system simulated at 280 K. **(C)** Shows the ρ_*L*_ of the AQP0-DMPC system at 310 K, where gel-phase was induced in the DMPC lipids (see Methods). **(D)** Depicts the AQP0-DMPC system simulated at 310 K, where the mobility of the non-hydrogen atoms of the protein was restrained using a force constant of 1000 *kJ*·*nm*^−2^·*mol*^−1^. Ordering of the MD–ρ_*L*_, was observed beyond the *S*2 annular lipid layer (*top-view*), where high density hexagonal acyl-chain packing (gel-like) is shown as blue hexagons.

We first addressed the localization of the annular lipids by comparing the changes in the total ρ_*L*_. In Figure 2 the lipid densities of simulations at 310, 280 K, under induced DMPC gel-phase, and with the protein restrained were compared. The ρ_*L*_ at 280 K (Figure 2B) and the DMPC-gel at 310 K (Figure 2C) displayed similar higher annular ρ_*L*_ at the extracellular side, and ρ_*L*_ was more localized at *S*2 than *S*1, compared to those of the unrestrained system at 310 K (Figure 2A). A similar ρ_*L*_ distribution was observed at 300 K (Aponte-Santamaría et al., 2012). In contrast, the simulation with the protein restrained showed a considerable increase of the annular ρ_*L*_ throughout the AQP0 surface (Figure 2D). The extent of this density increase made the annular ρ_*L*_ similar to those in EC (Figure 1B).

In order to test whether the ρ_*L*_ asymmetry between surfaces *S*1 and *S*2 was influenced by sampling, we increased the simulated time used to calculate the MD-ρ_*L*_ in the unrestrained simulation at 310 K by a factor of ten, from 280 ns to 2.8 μs (Figure S1). We observed that using 2.8 μs, the qualitative ρ_*L*_ localization was similar as for 280 ns. Nevertheless, by using 2.8 μs the ρ_*L*_ intensities decreased considerably mostly on *S*1. This result showed that annular lipids have a localization preference on the surface *S*2, especially at the extracellular side.

High ρ_*L*_ was also observed beyond the annular lipid layer in all conditions and mostly around the extracellular part of *S*2 (*top-view* perspectives in Figure 2). These ρ_*L*_s displayed the characteristic hexagonal packing of DMPC acyl-chains in the gel-phase (*L*_β_) (Smith et al., 1990; Akabori and Nagle, 2015). Gel-like lipids were expected in the 280 K simulation and in the induced DMPC-gel simulations. Nevertheless, these gel-like ρ_*L*_s were also observed in the unrestrained and in the protein restrained simulations at 310 K. Such hexagonal pattern of ρ_*L*_s included 4 acyl-chains of the highly localized annular lipids and extended clearly to several lipid layers as it is observed in the protein restrained and 280 K simulations. This evidence suggests that *S*2 induces local gel-phase changes in the surrounding membrane.

### 3.2. Lipids interactions and protein fluctuations

Higher annular lipid densities on the AQP0 surfaces are an indication of favorable interactions between the lipids and the protein surface. The lipid localization may be due to global interactions of the different components of the AQP0-DMPC-water system, or to specific interactions with amino acids on the AQP0 surface. These interactions might change with the DMPC lipids in the gel-phase. In addition, we wondered if the gel-phase transition of the lipids may lead to reduced protein mobility.

To understand the protein-lipid interactions, we estimated the global interaction energies of DMPC lipids with other DMPC molecules (self), with the protein, and with water. We also decomposed the lipid–amino acid interaction energies in vdW and electrostatic components, and mapped these values on the AQP0 surface. The results of both procedures are shown in Figures S2A–C. The small observed differences among the interaction energies, which account for the entalpic contribution to the free energy of the simulated AQP0-DMPC systems, did not explain the increased localization in the annular lipids when restraining the protein. This suggests that the reduction in entropy imposed by restraining the protein crucially contributes to the lipid immobilization.

**Figure 3:**
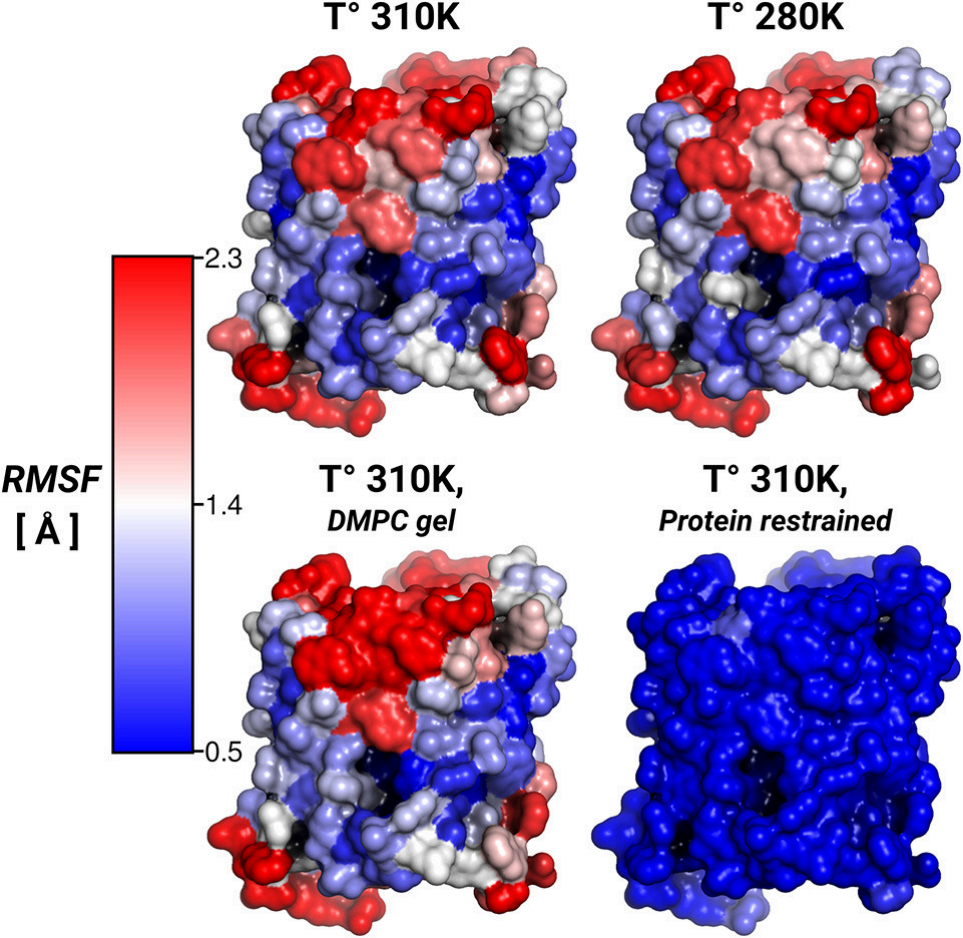
**Protein root-mean-square-fluctuations (RMSF)**. The figure displays the protein RMSF per amino acid mapped on the AQP0 surface. The RMSF color scale is shown on the left. The average protein, and backbone fluctuations of the simulations at 310, 280 K and DMPC-gel were ~ 1.7, and ~ 1.2 Å, respectively. The average fluctuations of the restrained protein simulation were smaller than 0.3 Å.

To address the lipid influence on the protein mobility, we calculated the root-mean-square fluctuations (RMSF) of the protein atoms, and mapped the amino acid contributions to the RMSF on to the protein surface. Figure 3 showed very similar RMSF profiles for the simulations at 310, 280 K, and for the induced DMPC-gel. These RMSF profiles showed that *S*2 was the more rigid surface of AQP0, followed by *S*1. On the contrary, the *S*1||*S*2 intra-monomer interface fluctuated more, especially at the extracellular side. These profiles were, as expected, very different to the restrained protein simulation, and suggested that the lipid mobility did not affect the protein flexibility.

Thus, while the restriction of the protein mobility was found to induce lipid ordering, the opposite was not found to be the case: inducing increased lipid ordering did not result in a measurable effect on protein flexibility.

### 3.3. Lipids ordering distribution

**Figure 4:**
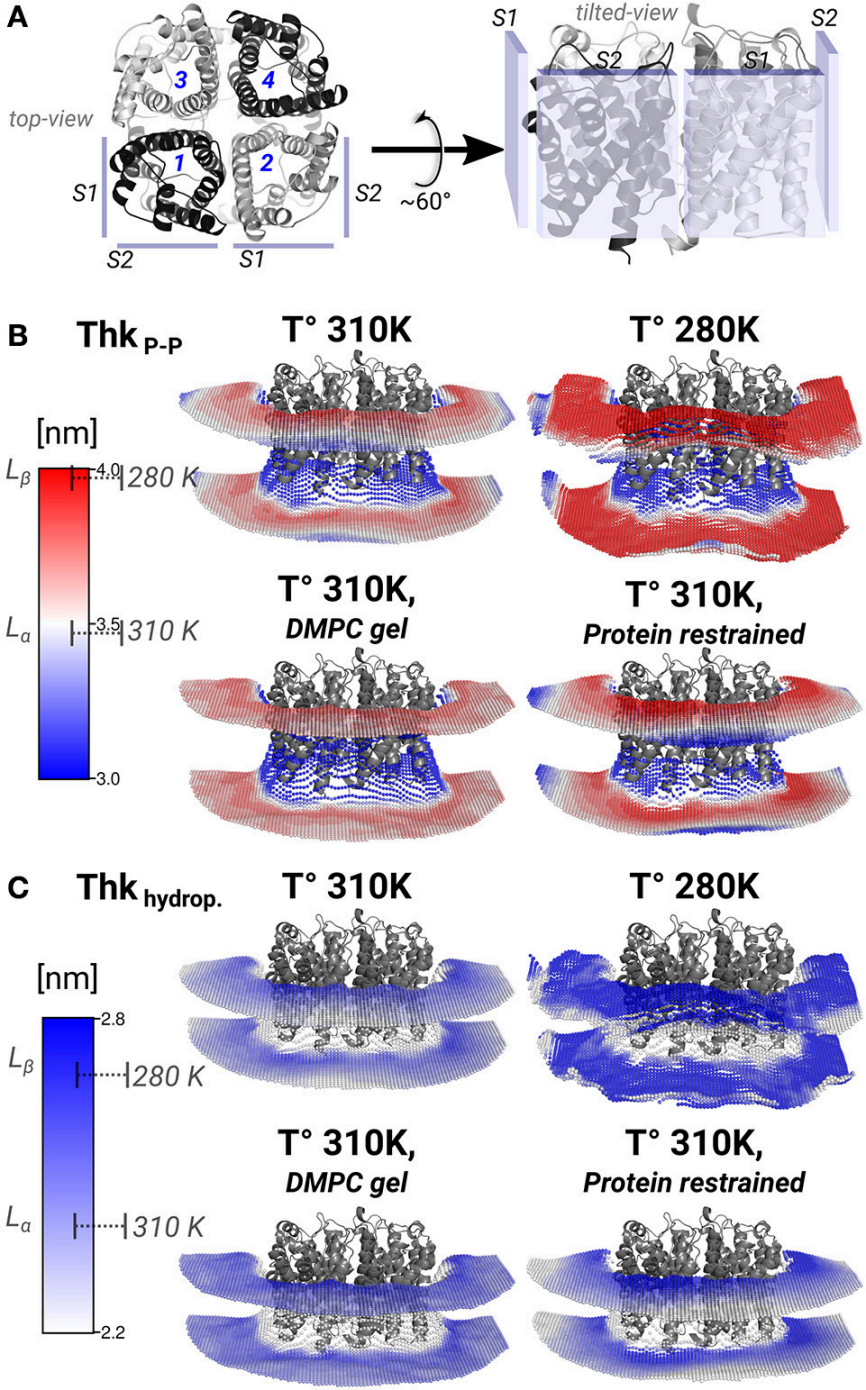
**Lipid thickening compensate the hydrophobic mismatch in the AQP0-DMPC system**. Local inter-phosphate (ThkP–P), and hydrophobic thicknesses (Thkhydrop.) profiles. **(A)** Depicts AQP0 monomers with numbers, and the surfaces *S*1 and *S*2 of two adjacent monomers, from a *top-* and a *tilted-view*. The *tilted-view* was used in the subsequent representations. **(B,C)** Show monomer- and time-averaged ThkP–P and Thkhydrop. around an AQP0 tetramer. The thicknesses values were calculated with g_lomepro (Gapsys et al., 2013) in a grid of 100 × 100 bins. Only the grid elements within 6.5 nm of the tetramer center-of-mass are shown. The scales on the left show the range of thicknesses spanned by the simulations. The segmented lines on the scales mark the average (± SD) values of a simulated pure DMPC patch at 310 K (fluid-phase, *L*_α_) and at 280 K (gel-phase, *L*_β_). The calculated thicknesses show that DMPC lipid thickening occurs close to the AQP0 surface at physiological temperatures. Annular lipid thickness values became close to those of gel-phase DMPC.

**Figure 5:**
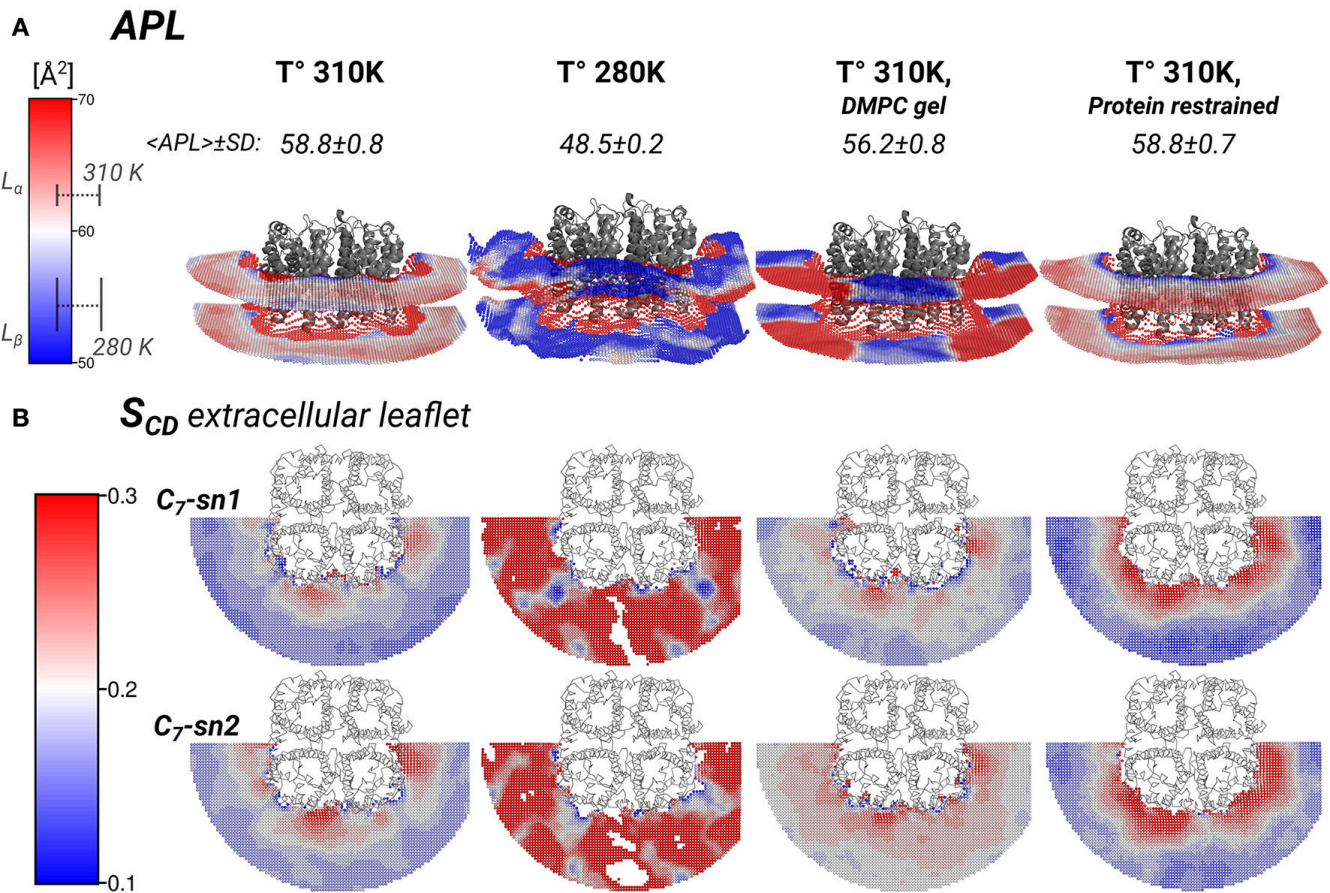
**Local area-per-lipid (APL), and SCD order parameters around AQP0 indicate phase prone specific subdomains**. Grid elements within 6.5 nm of the tetramer center-of-mass are shown. **(A)** Shows the calculated local and average APL. The color-scale shows the range of APL spanned. The segmented lines on the scale mark the simulated average (± SD) values of pure DMPC-water systems. **(B)** Shows the calculated SCD of the carbon-7 (C7) of both acyl-chains (*sn1* and *sn2*). On the left of the color-scale shows the value range from 0.1 to 0.3. Order parameters lower than 0.1 were omitted in the representations. Extracellular and intracellular SCD of acyl-chain carbons 2, 7, and 13 are shown in Figures S3, S4, respectively.

The lipid densities showed that the annular lipids localization was most affected by the protein mobility. There were also hexagonal acyl-chain patterns that included the annular lipids on *S*2, in all simulated conditions (Figure 2). These hexagonal patterns are a local sign of gel-phase ordering introduced on the lipids by an AQP0 tetramer. Gel- or fluid-phase lipid ordering might be observed in its local properties such as thicknesses, area-per-lipid (APL), and carbon-deuterium (SCD) order parameters (Vermeer et al., 2007). These calculated lipid properties were monomer- and time-averaged and, they might suggest the type of hydrophobic mismatch mechanism, the degree of lipid compactness, and the acyl-chain ordering, respectively. The inter-phosphate (P–P) and hydrophobic thicknesses showed that thickening of the annular lipids is the mechanism to compensate for the hydrophobic mismatch in the AQP0-DMPC system at physiological temperature (Figure 4). APL and SCD indicated the presence of specific gel- and fluid-phase prone areas around AQP0 (Figure 5).

#### 3.3.1. Lipid thickening compensate the AQP0-DMPC hydrophobic mismatch

Thickening of DMPC around AQP0 compensated the mismatch in the AQP0-DMPC system at physiological temperatures. Figure 4 shows the inter-phosphate (ThkP–P) and hydrophobic thicknesses (Thkhidrop) calculated around an AQP0 tetramer. Different monomers and the context of surfaces *S*1 and *S*2, are shown in Figure 4A. The thicknesses (Figures 4B,C) were mapped onto a grid, where the color indicated the thickness value, and the relative *Z*-height in each leaflet, represented the mean position of the atom(s) considered in the calculation (See Methods). For both thicknesses, the color-scale on the left included the average reference values of pure DMPC-water system simulations at 310 K (*L*_α_) and 280 K (*L*_β_).

The free and protein-restrained simulations at 310 K showed clear rims of higher ThkP–P and Thkhidrop that were in contact with the whole protein surface. These rims increased upon restraining the protein, and they showed slightly lower thicknesses values at the *S*1||*S*2 intra-monomer interface. The thickness values of the annular lipids became closer to those of gel-phase DMPC (280 K and induced DMPC-gel simulations), and showed that at physiological temperature DMPC lipids stretched to accommodate to the AQP0 surface. Beyond the annular lipids the ThkP–P at 310 K (free and protein–restrained) converged to their bulk values. In the simulations at 280 K and in the induced DMPC-gel, most of the lipids had homogeneous *L*_β_ DMPC thicknesses, as expected. This evidence suggests that surface *S*2 may accommodate longer lipid acyl chains, as indeed observed for *E. coli* polar lipids around AQP0 (Hite et al., 2010). This information indicates that the formation of AQP0 tetrameric arrays is possible under different hydrophobic matching circumstances. Furthermore, it suggests that the lipid accommodation observed here for DMPC may also occur for other more physiologically relevant lipid species beyond *E. coli* polar lipids.

#### 3.3.2. AQP0-DMPC system shows specific *L*_α_ and *L*_β_ areas

The thickness changes indicated the presence of *L*_β_-like annular lipids, and the lipid densities showed the presence of *L*_β_ acyl-chain packing by the *S*2 AQP0 surface. Those lipid changes might come also with changes in the APL, which reflect the global degree of lipid compactness. In addition, it might be associated with changes in the in SCD order parameters that report on the degree of ordering of acyl-chain carbons, which become higher in less mobile acyl-chains (Vermeer et al., 2007; Klauda et al., 2010). Both, APL and SCD might have different values on the extra- or intra-cellular lipid leaflets, and they have characteristic values in *L*_α_ and *L*_β_ phases. For that purpose, we calculated the local APL (Figure 5A) and the SCD (Figure 5B) around the AQP0 surface. As a reference, the SCD of pure DMPC patches at 280 and 310 K are shown in Figures S3A, S4A.

Local APL and SCD showed very similar phase-prone areas around AQP0. In the case of APL (Figure 5A), we found that the most compact (*L*_β_) lipid surfaces were those close to *S*2. In the free and restrained protein simulations at 310 K we found areas of low APL (*shades of blue*) near the protein surface. The presence of these areas was an indication of more packed lipids compared with the rest of the patch. These low APL areas coincided with the location of high annular lipid density, as it was observed in the extended low APL rim around the whole AQP0 tetramer in the restrained protein simulation. The average APL of both simulations converged close to their bulk values. As expected from gel-phase lipids, mostly low APL lipids were observed in the 280 K simulation case. Interestingly, the induced DMPC-gel simulation showed two distinct areas: one around the *S*2||*S*1 inter-monomer interface that showed low APL, and the *S*1||*S*2 intra-monomer surface, which showed higher APL and higher mobility (Figure 3). This APL evidence suggests that the less mobile parts of the protein are able to induce a more compact (gel-like) lipid domain, whereas the more mobile parts, are surrounded by more fluid-like regions. In addition, this evidence indicates that annular lipids are compactly packed around the AQP0 surface.

Similarly to the APL, the distribution of SCD showed two different phase prone areas. In the presence of AQP0, the SCD of the acyl-chains of the extracellular leaflet became more ordered compared to the intracellular one. Extracellular and intracellular SCD plots of carbons 2, 7, and 13 are shown in Figures S3, S4, respectively. As reference Figures S3A, S4A show the SCD profiles calculated on a DMPC-water system simulated at 280 and 310 K. As an example of the lipid ordering, the extracellular SCD of the carbon-7 from both acyl-chains are shown in Figure 5B. It became clear that the SCD were also locally affected by the changes in the protein mobility. In this case also, higher acyl-chain carbon ordering was observed mainly near the *S*2 surface, while lower order was observed by the *S*1||*S*2 intra-monomer surface. Lipids SCD approached to the DMPC bulk values, the further away they were from the AQP0 surface.

*In silico* mutations of the strongly interacting residues on *S*2 with DMPC (Aponte-Santamaría et al., 2012) did not substantially modify the lipid positions, and similar packed acyl-chain densities were always observed in the mutant simulations. This evidence suggests that a specific amino acids sequence on the *S*2 surface is not necessary to promote the gel-like packing of lipids in this region. Instead, we think, that the aquaporin fold, containing six tightly packed transmembrane helices, largely defines the rigid regions that are particularly effective to immobilize lipid molecules. Indeed, different aquaporins (sharing the same protein fold) displayed conserved lipid-protein interaction patterns (Stansfeld et al., 2013). Furthermore, we think the lipid distributions and ordering of a single AQP0-DMPC system are compatible with the observed AQP0 squared arrays separated by an ordered lipid layer.

## 4. Conclusions

In this work, we estimated the lipid localization and ordering under different temperatures, phases, and protein mobility. It was revealed that protein mobility largely dominates the annular lipid localization. Surprisingly, the opposite does not hold: lipid immobilization was not found to significantly affect protein mobility. The *S*2 surface of AQP0 was able to induce lipid localization in and beyond the annular lipid layer, also at 310 K. Local lipid properties indicated that thickening and ordering of DMPC annular lipids is the mechanism by which the AQP0-DMPC system compensates the hydrophobic mismatch at physiological temperatures.

The area-per-lipid and the S_CD_ distributions around AQP0 consistently showed two lipid subdomains, one of gel-like lipid properties close to the *S*2 surface, which propagated to *S*1 of the contiguous monomer, and the other of more fluid-like properties at the more mobile *S*1||*S*2 interface.

We showed that the AQP0-DMPC system —and probably AQP0 with other similar lipids— displays lipid subdomains of different localization and ordering. These areas might guide the interaction of AQP0 with other membrane components, including the self association of AQP0 copies.

The protein mobility may play an important role, not only for AQP0-DMPC interfaces but also for other systems in which proteins critically modulate the conformational dynamics of their surrounding lipids (Piknová et al., 1993; Niemelä et al., 2010; Stansfeld et al., 2013). Future efforts and progress, from the experimental and computational perspective, may help us to increase our understanding of the nanoscopic details of the lipid-protein interactions.

## Author contributions

RB, BD, CA developed the concept of the study. RB prepared the simulations and MD analysis. CA developed the MD density analysis tools and critically revised the manuscript. RB and BD wrote the paper.

## Funding

The Deutsche Forschungsgesellschaft (DFG) Sonderforschungsbereich (SFB) 803: Project A03 to RB and BD. This work was supported by grants from the Max Planck Society (CA and BD), the German Research Foundation to the Research Group FOR1543 (CA), and the BIOMS postdoctoral program of the Heidelberg University (CA).

### Conflict of interest statement

The authors declare that the research was conducted in the absence of any commercial or financial relationships that could be construed as a potential conflict of interest.
