# Supplementary material for: Localization and Ordering of Lipids Around Aquaporin-0: Protein and Lipid Mobility Effects
Source: Front Physiol. 2017 Mar 2;8:124. doi: 10.3389/fphys.2017.00124 (PMC5332469; doi:10.3389/fphys.2017.00124)
Supplement: Supplementary file 1 [file Image1.PDF]

Supplementary Material: Localization and ordering of lipids around Aquaporin-0: Protein and lipid mobility effects

Rodolfo Briones, Camilo Aponte and Bert L. de Groot

*Correspondence:
Bert L. de Groot
bgroot@gwdg.de

1 SUPPLEMENTARY TABLES AND FIGURES

1.1 Figures

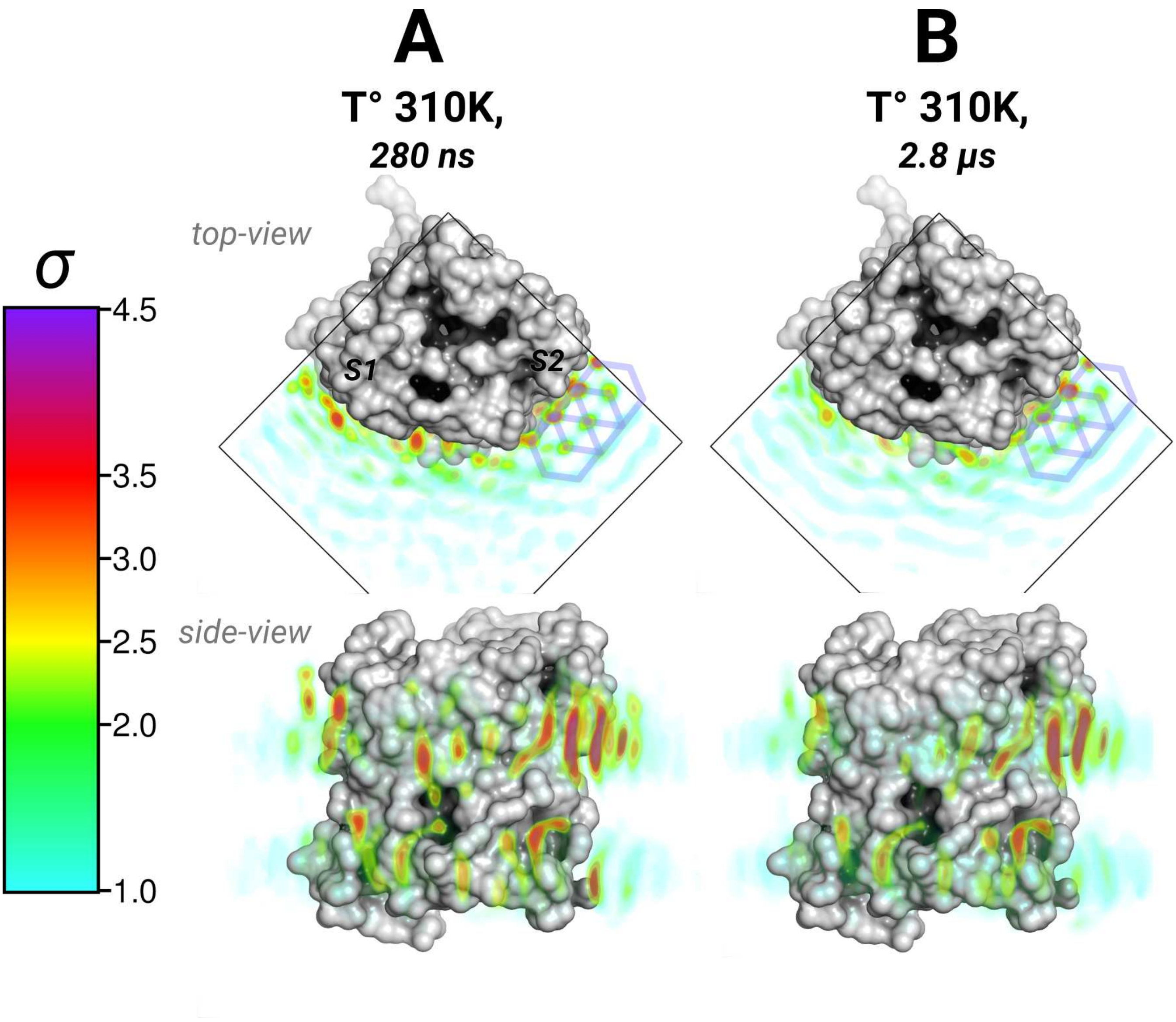

Figure S1. Time-averaged molecular dynamics (MD) lipid-density (ρ_L) sampling comparison. **A** and **B** show the normalized ρ_L calculated from unrestrained AQP0-DMPC MD simulations at 310 K. σ scale on the left. In **A** 280 ns were used to calculate ρ_L. In **B** 2800 ns were used to calculate ρ_L.

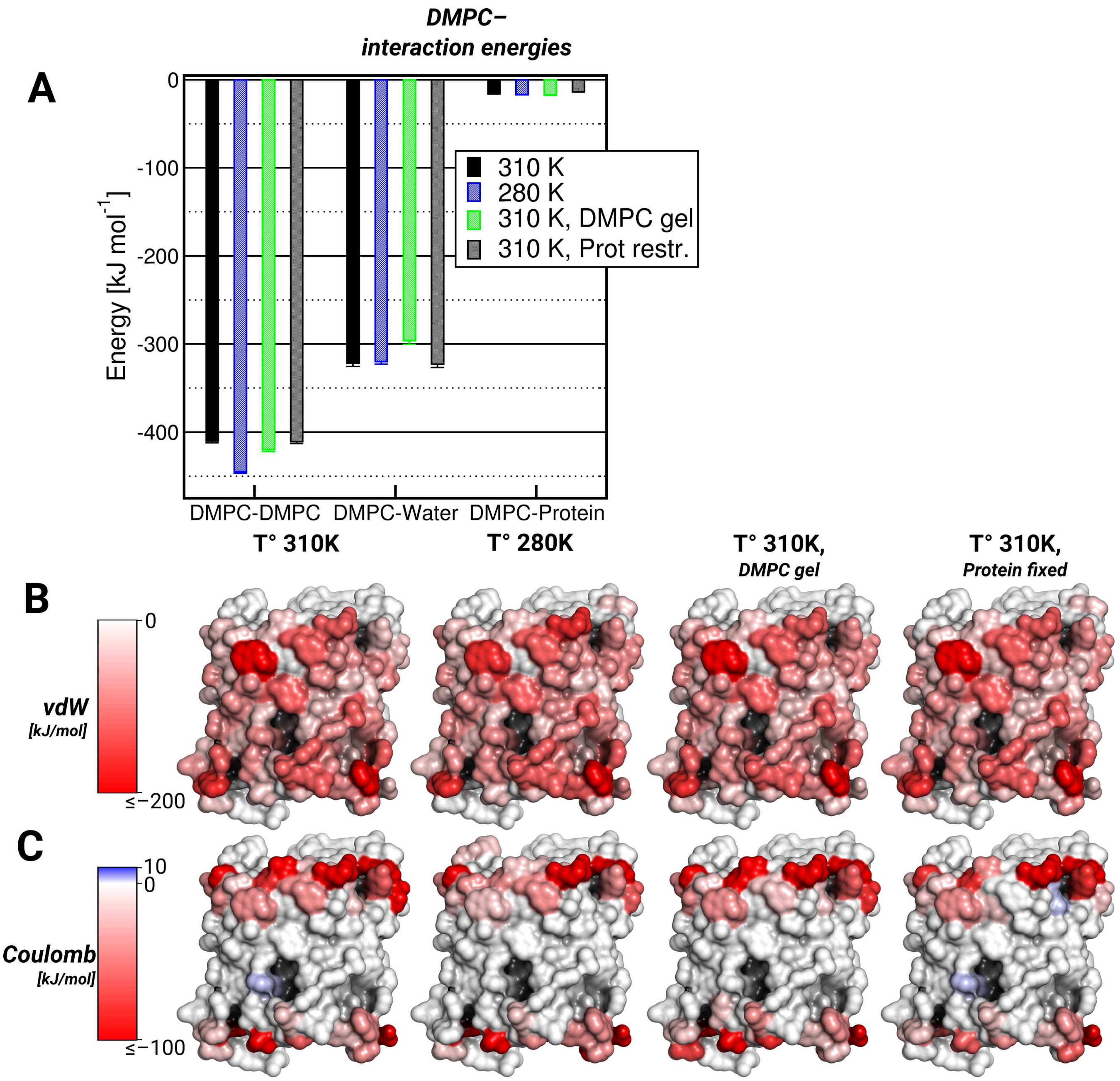

Figure S2. Global and local lipid interaction energy components. **A** shows the average (± SD) interaction energies of DMPC with DMPC (self), water, and protein. The energies were all attractive (negative sign), dominated by the lipid self interaction, followed by the DMPC-water, and a minor contribution was due to the DMPC-protein. When reducing the temperature from 310 K to 280 K the DMPC self interaction increased while the other terms remained similar. The induced gel-DMPC system decreased only moderately the DMPC-water energy, while leaving the self and the DMPC-protein components unchanged. By restraining the protein, the average energy components were indistinguishable to the unrestrained simulation at 310 K. **B** shows the lipid–amino acid interaction energies separated in van der Waals (vdW) and electrostatic contributions. The vdW and electrostatic interactions energies were similarly distributed over the surfaces $S1$ and $S2$ in all simulation conditions. vdW interactions uniformly covered the surface, while the electrostatic interactions were restricted to the extra- or intracellular edges of the protein surface.

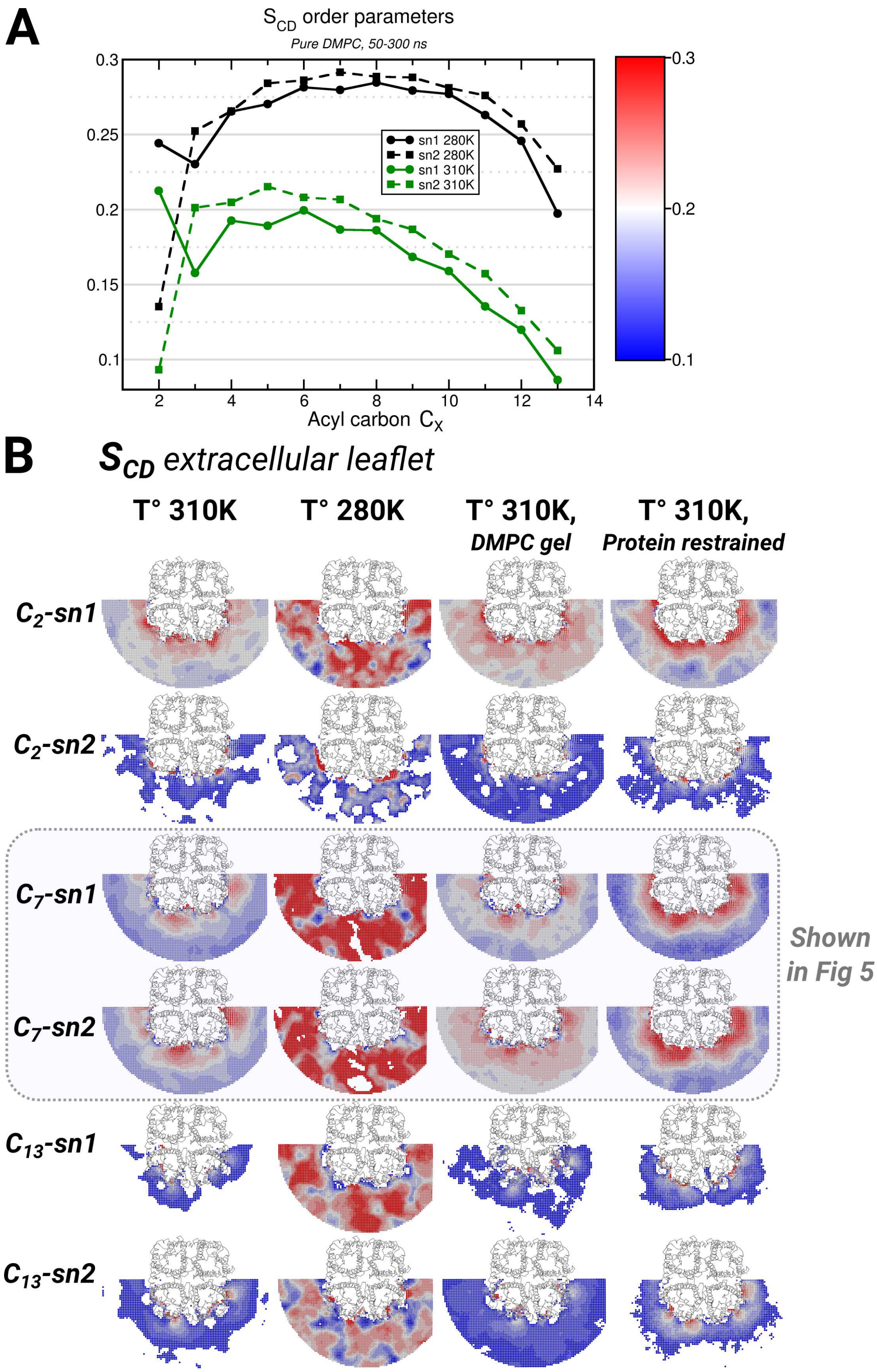

Figure S3. **S_{CD} order parameters of the extracellular lipid leaflet**. **A** shows the time-averaged S_{CD} of DMPC-water systems. The DMPC-water systems were simulated for 300 ns, excluding the first 50 ns as equilibration. The color-scale on the right indicates a range of order values from 0.1 to 0.3. **B** shows the extracellular leflet S_{CD} around AQP0 of acyl-chain carbons 2, 7, and 13.

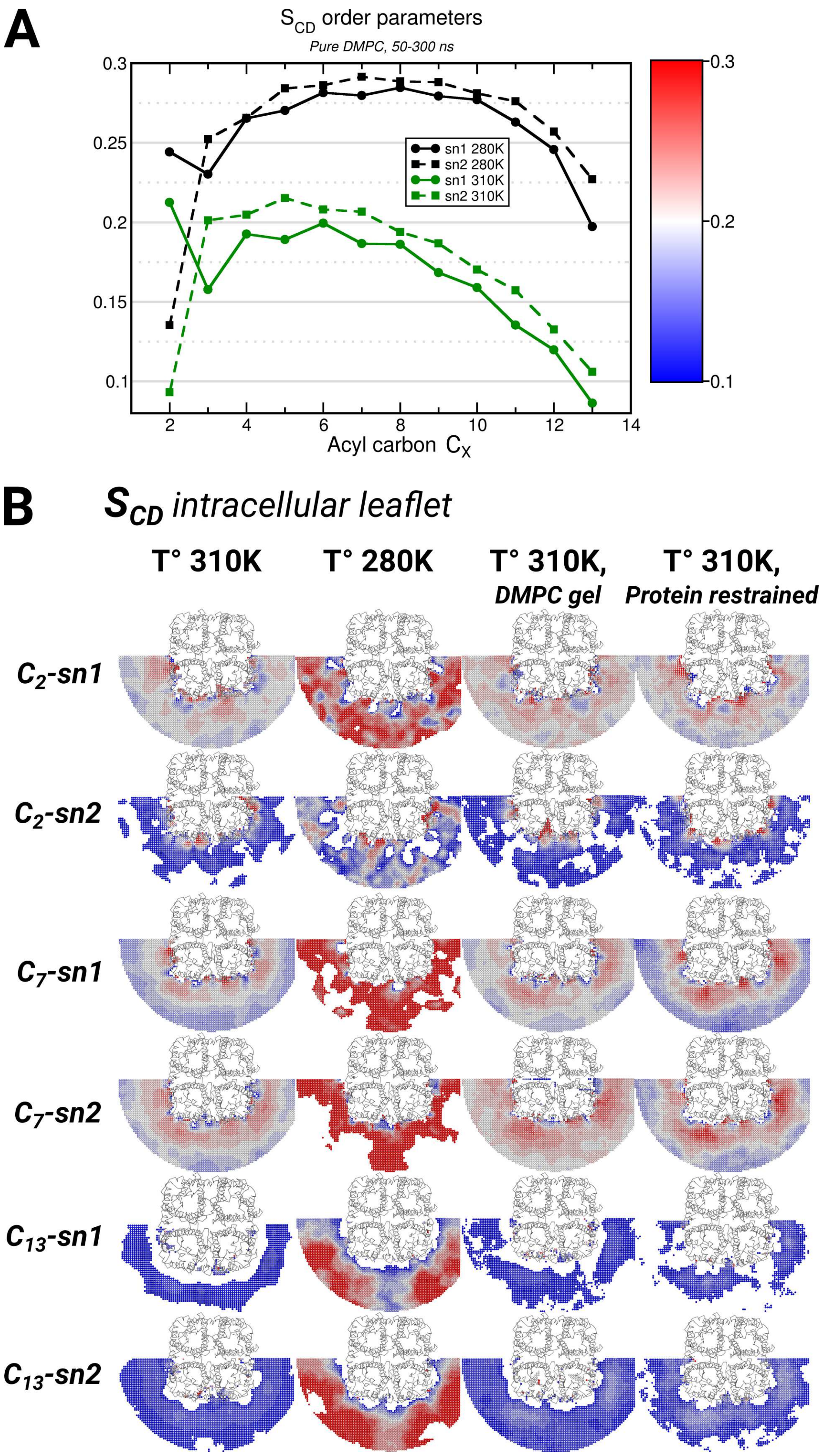

Figure S4. **S_{CD} order parameters of the intracellular lipid leaflet**. **A** shows the time-averaged S_{CD} of DMPC-water systems. The DMPC-water systems were simulated for 300 ns, excluding the first 50 ns as equilibration. The color-scale on the right indicates a range of order values from 0.1 to 0.3. **B** shows the intracellular leaflet S_{CD} around AQP0 of acyl-chain carbons 2, 7, and 13.
